# ‘Autism is the Arena and OCD is the Lion’: Autistic adults’ experiences of co-occurring obsessive-compulsive disorder and repetitive restricted behaviours and interests

**DOI:** 10.1177/13623613241251512

**Published:** 2024-05-10

**Authors:** Hannah Long, Kate Cooper, Ailsa Russell

**Affiliations:** Centre for Applied Autism Research, Department of Psychology, University of Bath, UK

**Keywords:** autism spectrum disorders, obsessive-compulsive disorder, repetitive behaviours and interests

## Abstract

Obsessive-compulsive disorder commonly co-occurs with autism. Research characterising the interplay between restricted, repetitive behaviours, activities and interests related to autism and obsessive-compulsive disorder symptoms has used theory-driven, bottom-up methodology. This study aimed to interview autistic adults about the subjective experience of differentiating between these phenomena. Semi-structured interviews were conducted with 15 autistic adults experiencing obsessive-compulsive disorder symptoms and repetitive behaviours, activities and interest. Transcripts were analysed using reflexive thematic analysis from a critical-realist, inductive orientation. Three overarching themes are presented. Participants viewed repetitive behaviours, activities and interest as intrinsic to their identity, while obsessive-compulsive disorder remained ego-dystonic and a perpetuator of anxiety. Conversely, repetitive behaviours, activities and interest was present across various emotions, often serving as a method to manage anxiety. Routinised behaviours and focused interests were considered by participants to be vulnerable to obsessive-compulsive disorder exploitation. Although participants reported masking both phenomena, the methods and motivations to mask differed. This research demonstrates the importance of delineating these experiences, with suggestions offered in how to explore this with autistic clients. Future research could explore narratives of masking obsessive-compulsive disorder across autistic and non-autistic people and investigate simultaneous co-occurrence of obsessive-compulsive disorder and repetitive behaviours, activities and interest; including how focused interests may influence obsessive-compulsive disorder and how repetitive routines may be intensified by obsessive-compulsive disorder.

**Lay Abstract:**

Repetitive behaviours and interests are a hallmark feature of autism. It is very common for autistic people to experience mental health difficulties, such as obsessive-compulsive disorder. Previous research has investigated similarities and differences between obsessive-compulsive disorder symptoms and repetitive behaviours in autism through questionnaires and observation studies. This is the first study to interview autistic adults about their personal experiences of differentiating between obsessive-compulsive disorder symptoms and repetitive behaviours related to autism. We interviewed 15 autistic adults who experience obsessive-compulsive disorder symptoms. We recorded these interviews and carefully analysed these to find themes. We found some differences between repetitive behaviours and obsessive-compulsive disorder. Participants said repetitive behaviours are part of who they are and what they want to be doing, whereas obsessive-compulsive disorder symptoms conflicted with how they view themselves. Obsessive-compulsive disorder was said to cause negative emotions, while participants said they experience lots of different emotions when doing repetitive behaviours. A similarity participants reported was trying to stop themselves from doing obsessive-compulsive disorder symptoms and repetitive behaviours that other people can see. There was also overlap between obsessive-compulsive disorder and repetitive behaviours. Participants talked about experiences when obsessive-compulsive disorder would take over routines and make them feel more intense and negative. Also, participants’ special interests were sometimes connected to the obsessions they experienced. We conclude that clinicians can use these findings to support conversations with autistic clients in differentiating between repetitive behaviours and obsessive-compulsive disorder symptoms. We also think that further research investigating how obsessive-compulsive disorder symptoms might be hidden by autistic and typically developing people is needed.

Approximately 1% of children receive a diagnosis of an autism spectrum condition (hereon in autism), although rates vary significantly across studies according to a range of sociodemographic and methodological factors (see Zeidan et al., 2022 for a review). Autism is characterised by social communication differences and a pattern of restricted, repetitive, stereotyped behaviours, interests, and activities (RRBI) (*Diagnostic and Statistical Manual of Mental Disorders*; 5th ed.; *DSM*-5; American Psychiatric Association (APA), 2013). DSM-5 denotes four sub-categories of the RRBI domain of autism: (1) repetition in motor movements, speech and use of objects, (2) insistence on sameness and inflexible adherence to routine; (3) abnormal focus and intensity on specific interests or hobbies; and (4) repetitive sensory sensitivity activities (APA, 2013). A wide range of heterogeneous phenomena are included within this domain from stimming behaviours such as hand or finger-flapping, to intense and focused interests on an object or topic area (F. Murray, 2019), and strict adherence to a routine that may cause difficulty with transitions or appear ritualised.

Research findings suggest individual experiences may not be adequately captured by such categorisation (e.g. Collis et al., 2022) and the terminology used in classification systems may not align well with language used by individuals themselves, reflecting deeper differences in the conceptualisation between clinicians, researchers and the autistic community. For example, the term ‘stimming’ is widely used by many autistic people (e.g. Kapp et al., 2019) with qualitative research describing physical movements which help to self-regulate, self-soothe and cope with conditions of boredom and stress (Collis et al., 2022). While similar in form to the phenomena labelled repetitive sensory motor behaviours (RSMB) in clinical classification systems and taxonomies, RSMBs have historically been defined as behaviours repeated in a consistent manner and appearing ‘functionless’ to an observer, highlighting differences in conceptualisation. A growing body of research involving autistic people and autistic researchers has led to a much richer and more valid understanding of underpinning mechanisms. The wide range of behaviours included in the domain termed RRBI and the heterogeneity across individual presentations of autism however adds greater complexity to establishing agreed definitions, terminology and conceptualisations.

An additional factor complicating the heterogeneous presentation of autism is the high rate of co-occurring mental health problems reported by many autistic people (Lai et al., 2019), particularly emotional disorders such as anxiety and depression. The lifetime prevalence of anxiety disorders for autistic adults is estimated at 42%, including obsessive-compulsive disorder (OCD) estimated at 22% (Hollocks et al., 2019). Findings across studies vary, for example, Lai et al. (2019) reported co-occurring, current OCD prevalence at 9%. Nonetheless, rates of OCD are consistently higher for autistic people when compared with non-autistic people. In a large prospective longitudinal study, Meier et al. (2015) found that a diagnosis of autism doubled the likelihood of also being diagnosed with OCD, and those with OCD were 4 times more likely to later receive an autism diagnosis; indicating a strong association between the two conditions.

OCD is defined as obsessions and/or compulsions which occupy more than 1 h/day (APA, 2013). Obsessions are understood as intrusive, persistent, and recurrent thoughts, images or impulses causing marked distress. Compulsions are behaviours or mental acts used to respond to or prevent imagined threat or harm.

There can be similarities in the behavioural topography of OCD and RRBI, leading to difficulties in delineating phenomenology and aetiology and identifying most appropriate support pathways. For example, ordering objects could be consistent with ‘just right’ compulsions as part of OCD or represent a preference for sameness in organising items of special interest as part of RRBI. Network analysis to investigate patterns of association between parent and clinician reports of autism and OCD symptoms identified distinct and separate symptom profiles for the two conditions (Ruzzano et al., 2015). Nonetheless, analysis indicated that compulsive or ritualistic behaviours, verbal rituals, and sensory interests were symptoms most likely to co-occur between autism and OCD, while obsessions were found to be the least inter-connected. These findings suggest that, while OCD and autism are separate conditions with different symptomatology, features can overlap across both conditions. However, a lack of subjective accounts and use of pre-defined symptom checklists are methodological issues which limit the conclusions that can be drawn from this study.

In addition to studies investigating similarities and differences at the level of behavioural topography, there have been efforts to understand the phenomenological experience of OCD and RRBI. This domain in autism has been associated with pursuing interests and pleasure (Turner-Brown et al., 2011) with the earlier mentioned qualitative findings adding nuance in that, for some individuals, these behaviours are an adaptive way of coping with negative emotional states (Kapp et al., 2019; Collis et al., 2022). Compulsions characteristic of OCD are associated with attempts to reduce significant distress (Morgado et al., 2013), primarily in response to distressing thoughts or images (Starcevic et al., 2011). OCD symptoms are consistently reported as ego-dystonic (Coimbra-Gomes, 2020; Meier et al., 2015) and dissonant with an individual’s values, needs and goals. In contrast, RRBIs are more often considered as harmonious with an individual’s sense of self; albeit this is not always the experience across the heterogeneous autistic population and within individual lives (Frost et al., 2019).

Research investigating efforts to hide one’s autism traits to counter stigmatising and dehumanising attitudes from others, known as masking or social camouflaging, has helped to elucidate further a more comprehensive understanding of the autistic experience (Pearson & Rose, 2021). Qualitative research findings report the suppression and substitution of a range of phenomena described in the RRBI domain (Collis et al., 2022; Kapp et al., 2019) suggesting that for many autistic individuals, these phenomena are not always experienced positively within a neurotypical society or in line with an individual’s needs and goals (Schneid & Raz, 2020).

Research investigating OCD symptoms co-occurring with autism have primarily used experimental, observational and cross-sectional study designs and quantitative measures, often parent and clinician symptom report. Some studies have reported reduced frequency of particular OCD symptoms, such as checking and cleaning (Scahill & Challa, 2016) and a less prominent experience of obsessions for autistic individuals (Zandt et al., 2007). In comparison, 95% of neurotypical individuals with OCD report contextually linked obsessions and compulsions (M. T. Williams et al., 2011). Other studies report findings of minimal differences between the two groups (Russell et al., 2005). This inconsistency in findings may in part be accounted for by differences in measurement methods and age.

The impact of OCD on autistic youth appears to be greater than neurotypical peers, with Martin and colleagues (2020) reporting poorer psychosocial functioning, longer service use, and smaller treatment gains. Research examining the effectiveness of Cognitive Behavioural Therapy (CBT) for OCD in autistic youth has shown that self-reported symptom improvement is significantly less compared with neurotypical individuals (K. Murray et al., 2015). However, clinical trials investigating CBT adapted for autistic adults with OCD have demonstrated effect sizes similar to those observed in trials involving neurotypical populations (Flygare et al., 2020; Russell et al., 2013).

Adaptations included emphasising the need to disentangle functional behaviours, often RRBI, from dysfunctional OCD symptoms in the assessment phase of treatment (Russell et al., 2019); without which treatment plans may mistakenly attempt to reduce otherwise helpful RRBIs. This would raise crucial ethical dilemmas as some RRBI can be experienced as part of someone’s autistic identity and can support self-regulation (Kapp et al., 2019).

In summary, this study underscores the frequent co-occurrence of OCD and autism and the clinical and conceptual issues relevant to identifying OCD. Distinguishing between OCD symptoms and phenomena included in the RRBI domain of autism is primary. The wide range of phenomena, heterogeneity in presentation, experiences and purpose, highlight the need for greater understanding and differentiation at phenomenological, construct and measurement levels. This will enable development and delivery of more tailored effective interventions. While OCD symptoms are well-defined, limited knowledge exists about the first-person subjective accounts of the distinct features of RRBI for autistic individuals, with most research relying on quantitative and observer-report measures.

In line with previous research aiming to understand the experiences of autistic individuals’ mental health (e.g. Goldfarb et al., 2021; Spain et al., 2020), RRBI (e.g. Collis et al., 2022) and the phenomenology of autism more broadly (D. Murray et al., 2023), this study uses semi-structured interviews and qualitative analyses to consider the question: how do autistic adults understand similarities and differences between RRBIs and OCD symptoms?

## Method

### Procedure

Ethical approval was obtained from University of Bath Psychology Research Ethics Committee and the study was pre-registered with the Open Science Framework.

Participants were recruited through purposive volunteer advertising using national charity networks and via a local volunteer database. Eligibility criteria were a clinical diagnosis of autism (including Asperger’s syndrome, Pervasive Developmental Disorder, ASD), current or historical OCD symptoms (no formal diagnosis required), >18 years old, English-speaking, no diagnosed intellectual disability or enduring mental health condition that significantly impacts functioning (other than OCD). The decision to exclude people with a diagnosis of intellectual disability was made on the basis that the research team did not have the full range of resources within the study to offer the communication or additional supports that might be potentially required to enable participation of this group. The study was not considered suitable for people with an enduring mental health condition in addition to autism and OCD as the study interview and question was a focused enquiry about disentangling RRBI and OCD and additional phenomena may introduce an additional source of variance for enquiry and analysis. Autism diagnoses were verified by viewing participant diagnostic reports and OCD symptoms were self-reported. Adverts led respondents to an online survey which included the participant information sheet, eligibility screening, written informed consent declaration, and demographic questions.

The Obsessive-Compulsive Inventory–Revised (OCI-R; Foa et al., 2002) was also used to characterise participants. The OCI-R is an 18-item self-report questionnaire assessing dimensions of hoarding, checking, ordering, obsessing, washing and neutralising on a 5-point Likert-type scale. Cadman et al. (2015) has demonstrated the OCI-R to have reasonable specificity and sensitivity across neurotypical and autistic populations, albeit a higher score of 29/72 is suggested to be more clinically relevant for autistic populations.

Eligible participants were contacted to arrange the interview with the first author, a neurotypical female clinical psychologist in training, and to consider what is required to facilitate cross-neurotype communication on an individual basis. The number of interviewees was decided during data collection by considering the richness and depth of the data and pragmatic constraints including timing and funding (Braun & Clarke, 2019).

The interview framework aimed to support participants to consider examples of RRBI, examples of OCD; how, when and what aspects of these phenomena overlap according to their experiences. Supporting materials were sent to participants beforehand to reduce memory-load, as is commonly reported by autistic individuals in interviews (Collis et al., 2022).

Interviews were conducted by the first author, took 60 to 90 min and were held between May and October 2022 either online or within a University research department according to participant choice. One participant requested a follow-up interview of 30 min. Common strategies to manage sensory needs and cross-neurotype communication included participants having their camera off during the interview, reduction of unnecessary noise and light, and seeking participants’ preferred language regarding autism beforehand. A range of visual aids were also available to support communication in interviews; in particular, a Venn diagram aided conversations about differentiation and crossover between the phenomena (see supplementary materials). Participants were asked what they might want to do following the interview, especially if they became distressed or uncomfortable. Answers were included in a debrief form as a reminder, alongside signposting relating to OCD support.

Interviews were audio-recorded and stored securely under password protection, only accessible by the research team. Interviews were transcribed using a systematic orthographic notation system (Braun & Clarke, 2013), before being safely deleted. Interview transcription was completed by either the first author or a professional service.

### Community involvement

Materials were developed alongside an autistic researcher with experience of completing qualitative research with autistic adults. They helped the first author/interviewer to consider cross-neurotype communication and interview conditions which enable trust and reciprocity (D. Murray et al., 2023). This led to the interviewer using the first point of contact with participants as a time to discuss individualising interviewer responses that might be more helpful for them; such as what the interviewer should look out for as signs of distress and how they would want this to be bought up by the interviewer if needed.

The autistic researcher also provided suggestions on how to adapt materials for participants and the development of the interview schedule and survey questions. An example of changes made from this was clarifying ambiguous phrases including removing degree adverbs, such as ‘very’.

### Participants

Demographic and relevant diagnostic data of the interviewed participants can be found in Table 1.

**Table 1.** Participants’ demographic and diagnostic information.

Variable	*n* = 15	Range (*M*, *SD*)
Current age	15	20–62 (*M* = 38.1, *SD* = 15.3)
Gender		
Female	6	
Male	8	
Other	1	
Ethnicity		
White British	14	
Black British	1	
Relationship status		
Single	8	
In a relationship	3	
Engaged	1	
Married	3	
Living situation		
Live alone	7	
Live with marital family	4	
Live with parents	4	
Employment^a^		
Volunteer	2	
Student	3	
Unemployed	5	
In paid employment	5	
Autism diagnosis		
Autism spectrum disorder	9	
Asperger’s syndrome	6	
Age at autism diagnosis	15	13–56 (*M* = 32.5, *SD* = 14.4)
OCD diagnosis		
Yes	10	
No	4	
Not sure	1	
Age at OCD diagnosis	10	13–58 (*M* = 26, *SD* = 13.6)
OCD treatment received	10	
CBT only	4	
CBT and medication	4	
CBT, medication, and hospital treatment	1	
None	1	
OCI-R total	15	20–64 (*M* = 38.6, *SD* = 11.8)
Within clinical range	11	
Other diagnoses		
None	4	
Depression only	4	
Multiple co-occurring diagnoses	7	

a If more than one, then the activity that takes up the most amount of time is stated here.

### Data analysis

Data analysis followed Braun and Clarke’s (2022) six-phase process for reflexive thematic analysis (RTA): data familiarisation, initial coding, theme generation, development and review of themes, refining and naming themes, and writing up. RTA offers flexibility in approach, allowing for inductively orientated experiential analysis focusing on patterns of meaning across the data corpus (Nowell, 2019). This process was embedded in a critical-realist standpoint which informed methodological choices allowing for transparency and complexity (Botha, 2021; Kourti, 2021).

The researcher read all transcripts to re-familiarise themselves with the data before coding. Semantic (descriptive, surface level) and latent (implicit) codes were generated through line-by-line coding. This was completed by noting codes alongside the transcripts within a table in Word. Fine-grained codes, focusing on detailed, micro-level meaning, were then copied into an Excel spreadsheet and grouped under overarching code labels according to semantic similarity. The integrity of these codes was reviewed by re-reading transcripts.

The researcher then developed themes by clustering overarching codes according to meaning or underlying central-organising concepts. To refine connections and boundaries between themes, visual mapping and summary paragraphs were used to draft central-organising concepts.

Quotes were chosen to represent the most concise and vivid examples that coherently exemplify theme meaning. The researcher aimed to use quotes from a range of participants without compromising clarity.

Participants’ preferred terminology was retained in the quotes (e.g. ASD or ‘special interests’), but in theoretical discussion and interpretation of the findings, we use the terms autism and focused interests in line with preferences for publication.

### Positionality

The research team was made up of three non-autistic clinical psychology researchers with experience of engaging with autistic young people and adults across a range of healthcare, educational and community settings. The interviews and analysis were conducted by the first author, a clinical psychologist in training, and discussed and developed with the other authors, both clinical psychologists and researchers. The first author kept a reflexive log (see supplementary materials for a summary) to consider the impact of their concurrent clinical psychology training on the practical and philosophical processes of the research (Trainor & Bundon, 2021; Braun & Clarke, 2023).

## Results

**Figure 1.**
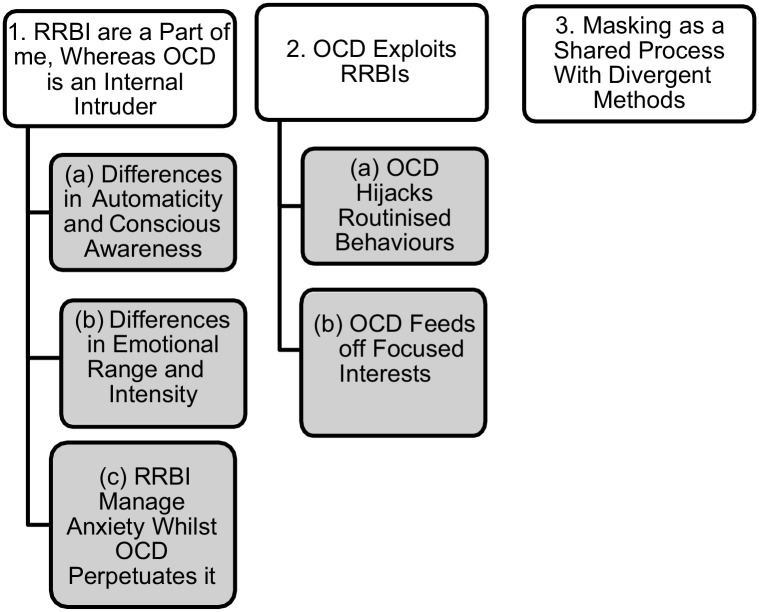
Themes and subthemes of the analysis.

The analysis resulted in three superordinate themes, with subthemes for the first two (Figure 1).

### Theme 1. RRBI are a part of me, whereas OCD is an internal intruder

Participants spoke about RRBIs related to autism being behaviours core and intrinsic to their identity. RRBIs were also thought of as useful methods to manage anxiety. In contrast, OCD was described as being incongruent with participant identity; yet participants spoke of an awareness of these experiences coming from themselves. OCD was discussed as confronting, unwanted, and both a cause and perpetuator of anxiety.

One participant summarised how OCD intrudes into the consistent and stable experience of being autistic with the following metaphor: ‘autism, you could say, is the arena and OCD is the lion. So, you’re in the arena and you’re already scared because something could happen and then OCD is the lion that’s happening’. (7)

#### Differences in automaticity and conscious awareness

Participants either explicitly described RRBIs as something integral and core to their personhood or implied this through their language use. An explicit example from one participant explained, ‘when I do it [stim], it sort of confirms who I am’ (13).

RRBIs were described as automatic behaviours that remain consistent with participants’ understanding of themselves. This automaticity is demonstrated in the following quote from a participant asked about how their RRBIs may have changed over time: ‘I don’t think anything particularly stands out because a lot of it [. . .] I don’t particularly notice because it’s just me. It’s just what I do’ (8). This quote suggests difficulty in remembering nuanced change due to RRBI being integral to their sense of self. Similarly, participants also noted that RRBIs aren’t experienced as time-wasting as they are intertwined with how they live: ‘the ASD ones don’t take up time because they are just part of how I live my life, so [. . .] they’re not really time-consuming things, they’re just how it is’ (11).

In contrast, participants’ described OCD as separate from how they see themselves. It was common for participants to speak about OCD coming from their brain/head/mind rather than from themselves: ‘just my head thinking’ (13)/‘my brain doesn’t accept it’ (9). Participants referenced that ‘the worse thing is [OCD] coming from yourself [. . .] you’re impacting yourself’ (4). This participant identifies the challenging recognition that OCD is coming from within.

Participants also discussed differing levels of effort required when making comparisons between RRBI and OCD: ‘I have to just work alongside [OCD], whereas the autism [. . .] is more intrinsic’ (14). This demonstrates the participant’s belief that OCD requires an active management process that is different to the automatic and intrinsic nature of autism.

Participants also spoke about compulsions having a thought driving them while RRBI happen without thinking:

(11): I think that the OCD stuff makes, I go into this mode where I’m being, what I know is being irrational, but there is some sort of twisted logic to it. [. . .] The ASD things, I think the difference is the absence of thoughts, because I just do them, I don’t really think about it and that’s the difference.

In this quote, the participant describes a ‘twisted logic’ and awareness of this ‘irrational’ way of thinking with OCD. This was a common thread across participants when discussing OCD, whereas behaviours related to autism were experienced in the moment without any accompanying thoughts.

Participants also spoke about feeling shocked, and a few people named feeling suicidal, due to their early experiences of OCD intrusions. Participants particularly related this to a lack of understanding about OCD: ‘The thoughts can generate- can make you go suicidal. When you first have them because you don’t understand what’s happening’ (3). This highlights both how dissonant OCD feels to participants’ sense of self, and how different this experience is to RRBI in which behaviours happen mostly without awareness.

#### Differences in emotional range and intensity

RRBI and OCD were both considered to be associated with difficult emotions, particularly anxiety: ‘There’s anxiety with both of them but not when I’m stimming but then there’s anxiety before I do it . . .’ (13). Here this participant names that anxiety is present for both experiences preceding the behaviour, yet during stimming there isn’t anxiety. Participants expressed that this was not the case for OCD: ‘There’s a lot more anxiety before and during the OCD stuff’ (11).

However, participants also named more diverse negative emotions with OCD than RRBI. Common emotions participants spoke of feeling with OCD were ‘fear’ (3/5/6/7/11/12/13/14), ‘distress’ (1/8/11/12/14/15) and ‘panic’ (5/8/14/15). Participants who described finding it hard to name emotions used other terms, such as ‘meltdowny’ (2), ‘intense [and] shocking’ (4) and ‘feeling detached’ (10). One participant expressed a more measured emotional reaction to OCD of ‘frustration [and] irritation’ (9). Nonetheless, there was an absence of positive emotion when discussing OCD across all participants.

In comparison, RRBI were spoken about as present across an array of emotional experiences:

(14): . . . the repetitive behaviours that come with autism, although some of them are related to anxiety, generally they aren’t particularly negative. So, for example, I have repetitive behaviours that I do when I’m happy or when I’m excited. I also have ones that I do when I’m anxious and not feeling the best, but I’d say it’s quite situational, [. . .] whereas with the things that are more obsessive and compulsive, it wouldn’t matter where you put me, I would still feel anxious regardless . . .

This quote suggests that RRBI are consistent across this person’s experience and present differently across emotional contexts; whereas, no matter the context, OCD was always accompanied by negative emotion.

#### RRBIs manage anxiety while OCD perpetuates it

Participants spoke about how RRBIs can be used to regulate emotional and sensory experiences. For example, the somatic experience of physical RRBI was described as the reason participants did the behaviours: ‘I think that’s the point of stims, it’s to use that physical sensation to help you and whether it’s [to] calm you down or regulate, self-regulate’ (11). This was a common experience with others commenting that ‘stims are a soothing mechanism’ (2).

Although primarily centred around stimming, routines and focused interests were also seen as methods to manage anxiety and sensory needs:

(13): so [RRBI are] obviously always worse, not worse but more intense, on a Wednesday especially because on a Wednesday I have quite a busy day [. . .] so I’m a lot more tense and so I stim a lot more and focus on my interests more.

As participant 13 denotes, there is not a singular RRBI method that manages anxiety, but instead they ‘are a series of tools that you put together to make your environment the way you want it’ (4).

In contrast, OCD was discussed as masquerading as a management strategy akin to RRBI; however, instead of resulting in an improvement in anxiety, OCD perpetuates an ongoing experience of intrusive thoughts and anxiety:

(4): I suppose repetitive behaviour wise the things you put in place make you feel comfortable. Obviously, the OCD kind of ritual you do makes you feel comfortable initially, so I suppose at that stage they’re similar, obviously it tails off and then it feeds the whole OCD thing, but at the initial stage I think they both make you feel more comfortable.

Participant 4 comments here that despite both RRBI and OCD initially appearing as methods to feel more comfortable, OCD ‘feeds’ into a perpetuating cycle of anxiety.

It was also noted that when OCD is present, RRBI such as stimming is used to manage the resulting anxiety:

(13): I also get quite restless if I can’t do [a compulsion] or if I’m in the process of doing it and it’s taking a while, so I will stim while I do it, like flap my hands which I know is related to my autism but then it links to my OCD as well because the OCD is causing the anxiety which causes me to stim.

This participant explains how RRBI, in this case stimming, can overlap with OCD, as the anxiety related to OCD prompts them to stim.

### Theme 2. OCD exploits RRBI

Participants described OCD as an active exploiter of RRBI. This theme considers how OCD intensifies the repetitive nature of routine and manipulates participants’ focused interests. Agency was often ascribed to OCD with terms such as OCD can ‘creep [around]’’ (4/8/14), ‘jump’ (4), ‘latch on’ (8) and ‘cling’ (7) to RRBI.

#### OCD hijacks routinised behaviours

Routines were highlighted as a similar feature of both RRBI and OCD. Participants considered how certainty and control are something that both autistic people and people with OCD seek:

(12): OCD wants you to be 100% sure about things and it wants you to not have any uncertainty in your life but then that’s also something that people with autism might experience. That they want certainty about things and structure and things not to change and that’s where I get a bit confused.

Alongside this commonality, there were many experiences across participants in which OCD hijacks or worsens autism associated routines:

(8): It’s part of my routine anyway to check when I get out the car. But then I think the kind of compulsion and like intrusive thought like feed into it and exacerbate it. [. . .] I think it worsens. It feeds into the need for it being repetitive.

This participant describes how intrusive thoughts take over an existing routine and increase the need for repetition. This quote suggests that OCD can be experienced as wanting control but not being able to be controlled. This is in comparison to how RRBI was discussed:

(14): Do I want to control [RRBI] necessarily? No, not always. But can I? Yes, I can. And I suppose on the other side of things, if I could control obsessions and compulsions then I most certainly would but that’s not something that I can do.

Both of the last two quotes speak to OCD being able to thrive in the context of wanting control and certainty within routine. They also highlight how OCD can exacerbate a want for routinised behaviours in a way that feels uncontrollable, while comparatively RRBI can be controlled even if this is not wanted.

This was further clarified when participants spoke about what might happen if routines were disrupted. Participants reported anxiety, frustration and negative self-perception in relation to feeling they must complete routines associated with either RRBI or OCD. However, disruptions to routines perceived as overlapping both phenomena were reported to cause significant distress (‘all hell break[ing] loose’ (7) whereas interruptions to RRBI-only routines were generally considered a ‘bother’ (9)). One participant’s response to being asked about what would happen if they didn’t complete their RRBI-only routines in comparison to RRBI routines hijacked by OCD was

(8): I feel really weird and anxious and particularly with the [RRBI only routines]. I just won’t feel right. [. . .] it’s more just feeling uneasy, like something that’s not right. I don’t particularly get into a meltdown over it, I just feel odd. [. . .] So, suppose–if it was like with the [RRBI+OCD routine]. I will go into a meltdown. It’s very easily- With my [RRBI+OCD routine] for me to go into a meltdown.

Here, this participant describes the intense emotional experience of not completing routines that OCD has hijacked from RRBIs as ‘meltdowns’. They denote a more nuanced emotional experience that is harder to articulate regarding not completing RRBI routines: ‘won’t feel right’, ‘uneasy’ and ‘odd’.

#### OCD feeds off focused interests

Participants spoke about their focused interests as being important to them, often engaging in these repetitively. Many noted how their core values often relate to these interests. Participants often reflected on how intrusive thoughts can intertwine with both their interests and values:

(14): I think [OCD] is quite closely linked with autism because my special interest [. . .] is medicine [. . .] I really like to care for people. I would never want to do anybody harm. I want to be a good person if I can. So, I kind of wonder if the reason I have these obsessions is because I’m so concerned about that.

Other behaviours that participants found difficult to differentiate began as an enjoyable activity related to participants’ interests, which then spiralled into OCD:

(11): With my interests, if something evokes an emotional response, makes me angry or upset or whatever, I can become very fixated on it and very interested in it. [. . .] That happens quite a lot with my interests; special interests are a good thing and generally people say that they’re just a positive thing, but they can have a darker side for me because I can get very obsessive and compulsive about them and then it becomes disruptive.

Participant 11 explains how OCD can increase ‘fixat[ion]’ on an interest and can turn something otherwise positive, into something disruptive.

### Theme 3. Masking as a shared process with divergent methods

This final theme considers participants’ narratives about masking overt RRBIs and OCD symptoms, particularly noted in relation to stimming and checking behaviours. Although masking appears to be present across both phenomena, the motivations and methods for this appear to be different.

Participants often spoke about how both began as physical actions that could be noticed by those around them:

(4): [OCD] very much started off as more physical manifestation [switching on and off] of the lights.

(11): I was rolling my top lip over and over again [RRBI] [. . .] Then someone in my class, I caught their eye and they stared straight at me and [. . .] made me realise clearly it was obvious to other people and, therefore, was a slightly unusual behaviour.

When participants discussed physical RRBI, social stigma was often considered as a reason to mask behaviours:

(14): I have to turn off certain behaviours and certain ways of thinking and doing, so that I’m able to function as I suppose what some of the population would say is normal. And that most of the time does mean trying my best to abandon repetitive behaviours, especially the physical ones because I feel like those come with the most stigma.

Comparatively, when participants considered how overt OCD behaviours changed over time, participants spoke of feelings of not wanting to interrupt or cause difficulties for those around them: ‘. . . like before other people would probably notice [OCD behaviours] but now no one would notice. No one knows. My family don’t realise anymore that I still experience it because it doesn’t affect anybody else (5)’.

Another difference between participants’ masking experiences was how behaviours are monitored and changed from overt to covert:

(13): Hand flapping is a lot more discrete [. . .] stimming you can hold it back and inwardly squeeze yourself and then release when it’s a more appropriate time.

(4): [OCD] morphed from more the physical checking and doing to the more visual. I guess maybe that was because I’d moved out, I was living with other people, and I suppose in a way it’s kind of masking the activity, but you still need the outlet somehow, so it became more visual then.

Both examples state active consideration and attempts to change or delay RRBI or OCD behaviours. Certainly, they appear to be different in methods, yet both state that a ‘release’ or ‘outlet’ is required for both behaviours.

Participants spoke about preferring physical manifestations of both behaviours:

(14): The pay-off of doing a physical [compulsion], [. . .] that for me has a larger impact on diminishing the obsession than would for, say for example, my mental checking.

(13): I always think that I look like a weirdo [when doing a physical RRBI] so then I need to stop doing it if I’m in public especially, but then the thoughts again are that it’s part of me so why should I stop.

Both quotes highlight that there is an advantage and desire to complete the physical form of both behaviours, albeit for different reasons.

## Discussion

The results of this study provide insights into the subtle and unique similarities and differences between RRBI and OCD according to the subjective experience of autistic adults. Across 15 semi-structured interviews, three themes were identified by the researcher using RTA (Braun & Clarke, 2022).

The first theme supports pre-existing understanding that autism and RRBI can be experienced as core components of oneself to the extent they are experienced as automatic and span the breadth of most emotional experiences, as well as RRBI being seen as useful tools to manage anxiety (Collis et al., 2022; Manor-Binyamini & Schreiber-Divon, 2019). Whereas, OCD is implied to be an internal intruder that is only ever experienced negatively and perpetuates anxiety (Elliott et al., 2021). Some participants in this study spoke about their experiences of obsessions as being both shocking and irrational, similar to descriptions of obsessions in neurotypical populations (Summerfeldt et al., 2014). This adds to ongoing debates regarding how and to what extent autistic individuals experience obsessions (Bedford et al., 2020). In the introduction, we wondered if accounts of masking and camouflage for RRBIs might reflect a dissonance in respect of the experience of these phenomena for individuals. Our findings weigh on the side of RRBIs being intrinsic to one’s sense of self and support the ego-dystonic nature of obsessions for autistic people, similar to the phenomenological accounts of non-autistic people.

The second theme reports OCD symptoms as exploitative phenomena that hijack and feed off aspects of RRBI, including routinised behaviours and focused interests. Particular notice is paid to how participants used language to personify OCD. Participants reported experiencing OCD as intensifying the need for repetition of routines and turning a potentially neutral emotional experience of RRBI into something negative. Focused interests were also noted to be incorporated into obsessional content. These findings are interesting alongside Ruzzano and colleagues (2015) results suggesting that aspects of autism appeared to precede and lead to OCD symptoms, such as sensory interests leading to OCD checking. This would be an interesting topic to explore in greater detail across different cohorts of autistic individuals.

The final theme captures the similarities and differences in masking related to both OCD and RRBI. Results regarding how and why participants mask RRBI were similar to previous research, including turning visible RRBI into covert behaviours to prevent others noticing due to social stigma. For example, both Collis et al. (2022) and Kapp et al. (2019) delineated participants’ narratives of supressing and substituting RRBIs as their search to be seen as ‘socially acceptable’, while feeling conflicted due to also wanting to express and accept RRBI. This study also considered how participants might mask RRBI, namely inwardly squeezing themselves to delay or placate urges. There is limited research investigating masking OCD symptoms and it would be useful to review if these experiences are comparable to neurotypical communities.

### Clinical implications

Russell et al. (2019) highlights the importance of a thorough assessment delineating RRBI and OCD with autistic clients to inform treatment. This research can contribute new perspectives to these conversations and influence design and development of more valid and reliable measures for assessment and differentiation of symptoms (Bedford et al., 2020). The emotional experience, before, during and after phenomena is highlighted as key to distinguishing OCD symptoms from RRBI. However, given the widely reported difference in emotional awareness that can be associated with autism (Huggins et al., 2021), including differences in interoception (Z. J. Williams et al., 2023), carefully scaffolded enquiry using visual cues to gather information about timing and nature of the emotional experience is clinically indicated as part of OCD assessment with autistic people. It may also be useful for clinicians to attentively listen for externalising and personifying language that may relate to ego-dystonic experiences of OCD. For example, clinicians may consider enquiring about overlapping experiences where RRBI might be hijacked by OCD and aim to understand what an individual’s usual experience of RRBI is versus what is new and ‘other’ in respect of current RRBI.

Evidence that OCD and RRBI are distinct as phenomena, but the co-occurrence can influence presentation (Bedford et al., 2020) is in part validated by participant accounts in this study. The ‘hijack’, ‘exploitation’ or ‘contamination’ of RRBI by OCD symptoms may mean that the form and intensity of the experience of the RRBI will change. This might involve avoidance, increased repetition or duration of a behaviour, or changes in usual form. This is different to understanding OCD compulsions as a relatively ‘new’ behaviour or occurrence for an individual, that is, something they did not need to do before.

The current research adds to ongoing narratives about stigma and societal expectations of the autistic population leading to masking not only autism characteristics but also OCD symptoms. These conclusions add further support for investigation into preventive strategies to disassemble stigma across society (Miller et al., 2021).

### Strengths and considerations

This study has significant strengths, including providing a valuable opportunity for autistic individuals with OCD to share their experiences and offer unique insights. A clinically appropriate level of support and materials were used, resulting in meaningful interviews which have been documented transparently for readers to adopt into future clinical and research opportunities.

A notable consideration includes the recruitment methods of this research, relying on specific diagnosis-based charities and networks. This may have meant that the recruited participants align more closely to these labels than others, potentially limiting the range of perspectives obtained. A more diverse recruitment approach, such as advertising at community centres or non-specific social media platforms, may be appropriate for future research. Similarly, future research may look to employ exclusion criterion that evaluate the level of adaptations necessary for each individual, rather than inferring elevated needs based on specific diagnostic labels.

The participants involved in this study were a diverse group to the extent that OCD had various degrees of impact on their lives. It was also interesting that most participants were diagnosed with OCD prior to autism. Meier and colleagues (2015) longitudinal cohort study suggested that associations between OCD and autism diagnoses were stronger for individuals with less obvious or intrusive autism characteristics, and without diagnosed intellectual disability. It would be important for future research to explore the experience of autistic people with intellectual disability to understand the relevance of the present findings. It is plausible that differences in the experience of RRBI as well as the degree to which individuals can influence and exert control on environmental circumstances may be important contextual factors.

## Conclusion

Semi-structured interviews and RTA methods (Braun & Clarke, 2022) were used to consider the personal experiences of 15 autistic adults in how they differentiate OCD and RRBI. This study is situated alongside qualitative studies investigating RRBI and various mental health conditions with autistic populations. The results of this study compliment previous findings, particularly in how participants make sense of their experiences by separating out ego-dystonic experiences of OCD against RRBIs which were talked about as an integral part of their everyday life.

This study also extends findings relating to masking RRBI and begins to consider similarities and differences of masking OCD symptoms. A finding that warrants further investigation in future research is the overlapping experiences of OCD and RRBI, such as how focused interests may feed into OCD and how repetitive routine may be intensified by OCD.

## Supplemental Material

sj-docx-1-aut-10.1177_13623613241251512 – Supplemental material for ‘Autism is the Arena and OCD is the Lion’: Autistic adults’ experiences of co-occurring obsessive-compulsive disorder and repetitive restricted behaviours and interests Supplemental material, sj-docx-1-aut-10.1177_13623613241251512 for ‘Autism is the Arena and OCD is the Lion’: Autistic adults’ experiences of co-occurring obsessive-compulsive disorder and repetitive restricted behaviours and interests by Hannah Long, Kate Cooper and Ailsa Russell in Autism
