# Supplementary material for: ‘Autism is the Arena and OCD is the Lion’: Autistic adults’ experiences of co-occurring obsessive-compulsive disorder and repetitive restricted behaviours and interests
Source: Autism. 2024 May 10;28(11):2897–908. doi: 10.1177/13623613241251512 (PMC11497754; doi:10.1177/13623613241251512)
Supplement: sj-docx-1-aut-10.1177_13623613241251512 – Supplemental material for ‘Autism is the Arena and OCD is the Lion’: Autistic adults’ experiences of co-occurring obsessive-compulsive disorder and repetitive restricted behaviours and interests [file sj-docx-1-aut-10.1177_13623613241251512.docx]

Supplementary material

Appendix A: Semi-Structured Interview Schedule

**Interview Topic Guide**

Investigating obsessive compulsive disorder (OCD) symptoms and repetitive behaviours in autistic adults.

Photograph of Interviewer redacted

This is a picture of me, Hannah Long. I will be the person interviewing you.

If you have not used Microsoft Teams before, please follow this link and read through how to access the call as a guest:

<https://support.microsoft.com/en-us/office/join-a-meeting-without-a-teams-account-c6efc38f-4e03-4e79-b28f-e65a4c039508>.

If you have Microsoft Teams downloaded but you are not sure how to access the call, please read the following link:

<https://support.microsoft.com/en-us/office/join-a-meeting-in-teams-1613bb53-f3fa-431e-85a9-d6a91e3468c9>.

Please let me know if you have any questions or problems leading up to the interview by emailing me ([hmrl20@bath.ac.uk](mailto:hmrl20@bath.ac.uk)).

The following is a topic guide meaning that the questions may not be stuck to rigidly. The questions are supposed to be broad and may overlap and so they can be asked flexibly depending on how you answer. We aim to have answers to all questions by the end of the interview. This has been sent to you ahead of the interview so that they can think about your responses in advance if you think this might support you in the interview.

Please let me know using the contact email at the top of this page if you have requests that would make your experience of the interview more comfortable and I will try my best to accommodate to your needs. For example, whether you would like my camera to be on or off, and I will try my best to accommodate to your needs.

**Introduction:** Thank you for agreeing to take part in this research interview. This interview should take approximately 45-90 minutes, depending on how much you would like to say.

Were you able to read through the supporting materials ahead of this interview including the participant information sheet you saw in the screening survey? Would it be helpful to read this again now before we start? I can share my screen now with the information if you haven’t got this.

Please could you now show me your diagnostic letter or report confirming your autism spectrum condition.

We will be talking about your experiences of autism and obsessive-compulsive symptoms and how these experiences fit together for you. You do not need to talk about anything you don’t want to discuss. It is very kind of you to speak to me about your experiences. As you know, this is a research interview and so how I answer or respond during the interview may feel different to other settings, such as an assessment for therapeutic support.

If at any point you feel upset or worried and you would like to stop the interview, then please do let me know. There will also be time at the end of the interview to discuss any questions or worries you might have.

During this interview, I might suggest we use some visual aids that we can look at together. These were included in your initial pack along with this topic guide. Would you like me to suggest times when we could use these during the interview or would you prefer me to not use these materials?

Do you have any questions before we begin?

Can I ask you to confirm for the audio recording that you fully understand what you have read about this study, that you are free to withdraw at any stage during this interview and that you agree to take part today?

I have to save this audio recording separately for anonymity reasons and so will stop this recording now and re-start a separate recording.

*Text in italics represents prompts/topics that may be used by the researcher if more information is required.*

1. What words would you prefer for me to use when talking about autism?

Please note that your answer to the first question will impact on how I change the wording of the following questions by using the terms you prefer to replace ‘autism’.

Before I ask you questions related to autism and OCD, I would like to know more about other aspects of your life. This is so that I can understand more about the people we speak to. It’s up to you if you would like to answer them or not.

1. Please tell me about any work or volunteering you currently do? If you do not work or volunteer, please tell me about what you do in a typical day.
2. What is your relationship status currently?
3. What is your living situation currently? Do you live with other people?
4. Do you have any co-occurring diagnoses that is not including autism or OCD? If yes, please tell me what these are.

Autism has been understood as differences in social communication and a pattern of restricted, repetitive behaviours, interests or activities. This interview is about your experiences of restricted, repetitive behaviours, interests or activities and symptoms you experience of OCD.

1. Please could you tell me about all the repetitive behaviours, routines, interests or activities that you engage with.

- Please could you now choose one particular repetitive behaviour we could talk about in more detail. Tell me as much about it as you can.
- *What feeling(s) do you notice when you engage in this behaviour or activity?*
- *What physical sensation(s) do you notice when you engage this behaviour or activity?*
- *What thought(s) do you notice when you engage in this behaviour or activity?*

Thank you for telling me about that in detail. Now I would like us to go back to think about all of the repetitive behaviours that you have mentioned earlier.

1. Have these developed or/and changed over time? How if so?

- *What are your earliest memories of repetitive behaviours, interests or activities?*
- *Have your thoughts/feelings/ physical sensations related to repetitive behaviours, interests or activities changed over* *time? How if so?*

I would like to know more about your experiences of obsessions and compulsions. You will have read about how obsessions and compulsions are characterised in the participant information sheet briefly.

1. What are your experiences of obsessions and compulsions?

- *Do you have a recent example of a compulsive behaviour that you could tell me about?*
- *What feelings, physical sensations and thoughts did you experience during this?*
- *Do you have a recent example of an obsessive thought or image that you could tell me about?*
- *What feelings, physical sensations and thoughts did you experience during this?*

1. Have the obsessions and compulsions you experience developed or/and changed over time? How if so?

- *What are your earliest memories of obsessions and compulsions?*
- *Have your thoughts/feelings/ physical sensations related to your obsessions and compulsions changed over time? How if so?*

You have told me about your experiences of repetitive behaviours, interests and activities related to your autism and also about your experiences of obsessions and compulsions. It will be helpful to hear about how you tell the difference between repetitive behaviours and OCD symptoms.

1. How do you tell the difference between repetitive behaviours and OCD symptoms?

- *In what way do you experience these as similar?*
- *Or different?*
- *How do they make you feel emotionally?*
- *How do they make you feel physically?*
- *How do you think?*
- *How do you behave?*
- *How do you understand their purpose?*
- *How much time do they take?*
- *When did they start in your life?*
- *When do they occur in the day?*
- *How are they similar or different before, during or/and after they occur in the day?*

1. How difficult is it for you to tell the difference between experiences of repetitive behaviours, activities and interests related to autism and obsessions and compulsions? Please provide a rating out of 10.

0 = Not at all difficult, can always tell the difference without needing to think about it

10 = Extremely difficult, can very rarely tell the difference and can take a long time to realise or understand

- *Is there times when it is not possible to differentiate between these experiences, please can you tell why this is?*
- *How do you feel if you are not able to differentiate between these experiences? Why is this?*

Thank you for answering my questions, that is now the end of the interview. Do you have any questions or concerns that you would like to ask me at this point?

I will send you a debrief form to your email address when we leave this call. This includes your pre-completed box of reminders of ways you thought you could look after yourself following this interview and some other avenues of support.

I will be sending your Love2Shop voucher for £20 in an email to you. Please confirm you have received this by sending confirmation to [hmrl20@bath.ac.uk](mailto:hmrl20@bath.ac.uk). This is very important and I will be very grateful to ensure you have received this.

Just to remind you, I will be anonymising what you have said when I type it up. This will be completed within the next 2 weeks. If you want to withdraw your data for any reason from this study, please make sure to contact me within these 2 weeks. Does that make sense?

Please do email me if you have any further questions in the future and thank you again.

Appendix B: Venn Diagram Visual Aid

Investigating obsessive compulsive symptoms and repetitive behaviours in autistic adults. These tools will be used adhoc by the researcher conducting the interviews to aid joint understanding.

Repetitive behaviour, interests, and activities

Obsessive compulsive symptoms

Appendix C: Researcher Positionality Statement and Reflective Log Summary

Researcher Positionality Statement

The first author is a novice to qualitative research and is currently training to become a Clinical Psychologist, working across various mental health settings with a particular interest in supporting neurodiverse clients. She is a neurotypical woman with personal and professional relationships with people experiencing OCD. The current research was developed by considering questions important to the autistic community (Autistica, n.d), relevant to clinical psychology and of interest to the authors. During the development of the research, the first author sought out clinical opportunities to work with autistic adults with OCD.

Reflective Log Summary

As first author, I kept a reflexive log spanning the entirety of the research process. Entries began in January 2022 when materials were being developed ahead of recruitment and ended in May 2023 during the editing phase of writing up the research for my doctoral thesis submission. Most log entries were a short paragraph reflecting on thoughts relating to the research that day, with some voice note entries. Key themes from the entries are summarised here.

Many initial entries focused on tension between putting lots of information into supporting materials defining OCD or RRBI. Feedback from an autistic qualitative researcher (Dr Emma Collis) during the development phase recommended some brief information, particularly about OCD symptoms. This was especially in light of there not being eligibility criteria for a formal diagnosis of OCD. However, I was resistant to putting too much leading information in about theoretical and diagnostic understanding of these phenomena as I wanted to hear from participants about their own unique ways of understanding these. This was an interesting contention between the medical model and positivist views that there is one true experience of these phenomena, and the epistemological angle of this research wanting to hear about how participants made sense of their own experiences from their own contexts.

During the first few interviews in particular, I often talked about feeling uncomfortable in a researcher role in reflexive log entries. This usually was due to how I am much more familiar with collaboratively formulating alongside clients, including with autistic individuals with OCD. The experience of having to follow the script and not interpret or provide summaries to clarify understanding was difficult for me. I also noticed I wanted to read up on the demographic information of the individual prior to the interviews. To prevent bringing my own framework of understanding to interviews, I followed my supervisor’s advice about sitting back within interviews and having clear prompting questions. We also added in a scripted explanation to participants that this interview would be different to clinical settings. This helped me feel more at ease by clarifying expectations of the interview, as well as reminding me about the stance I needed to take within interviews.

A-prior methods to support participants’ potential distress during the interviews were also discussed in my logs and taken to supervision. My supervisor was part of a RCT study alongside my project. We requested the use of their distress protocol and adopted this into our ethics application. As part of this, and alongside recommendations from Dr Collis, I also discussed reasonable adjustments with each participant ahead of interviews, including how they would want to manage any understandable distress that may arise. This was necessary and useful to agree ahead of time; with three or four participants opting for a break within the interview process. No further action was required, and this was always contained within the interview. Several participants also commented on how valuable it was to think ahead of the interview about what they wanted to do directly afterwards as a soothing activity.

Across the recruitment process, several entries described my shock in how many participants were coming through so quickly. We had planned for a potential slog and several recruitment drives, meaning I was hugely relieved that we were wrong! I often reflected about how resilient and determined the participants were to explain their unique position and both the good and the bad of their experiences.

Several participants were keen to provide further information alongside their interviews by emailing me afterwards with heartfelt poems and requesting follow up interviews. This led to updating our ethics agreement allowing me to provide follow up interviews if participants requested this, or if they appeared to want more time than the 90 minutes initially suggested. I particularly noted how important it felt for participants to have their perspectives heard and that they wanted to represent themselves fully and accurately. This may also be integrated into their experiences of needing to explain themselves just right, as is common with OCD and autism. I certainly felt both honoured to hear their narratives, and aware of the responsibility I had to get this right for them.

I noticed that this also affected me later in the coding process. I often experienced thoughts such as: ‘if I move away from the nuance in the data, I might misrepresent participants’ experiences’. I quickly found myself buried in fine-grained codes. I used supervision often to review my coding and moved through the iterative process recommended by Braun and Clarke (2022) of clarifying codes and themes by going between the transcripts, codes, overarching codes, and themes.

During coding, my supervisor also picked up at the very initial stages that I was using coding labels that were influenced by my CBT-training. I had chosen to start the coding process with a transcript I felt I understood well, as I believed this might be easier. In supervision, we considered that the participant I had chosen to code first had recently had CBT for OCD and was using similar terminology at points to discuss their experience. Therefore, I was naturally reading between the lines, instead of staying close to the data. It was very helpful to have had this learning and redirecting early in the coding process and enabled me to stay closer to the data. I returned to re-code this transcript afresh later once I’d had more experience, and instead chose one that had felt particularly different to my way of conceptualising OCD.

There were a lot of logs during the theme generating and refining processes. Often these logs noted times when I had strayed away from the data too far and required redirecting back to the initial codes and transcripts. I also found I needed a lot more breaks during this time and particularly appreciated time spent discussing the boundaries of potential themes with my peer and good friend**.**

## References

Autistica (n.d.). *By 2030 autistic people will have proven treatments for anxiety*. Retrieved from: https://[www.autistica.org.uk/about-us/2030/2030-anxiety](http://www.autistica.org.uk/about-us/2030/2030-anxiety)

Appendix D: Acknowledgements

The authors thank Dr Emma Collis for her advice regarding participant accessibility of materials and interview schedule. We would also like to thank the Centre of Applied Autism Research, Autistica, the National Autistic Society, OCD-UK and OCD-Action for their support in recruitment for this project. We are very grateful to all participants who took part in the interviews and shared their experiences and views.
